# Delivery of integrated infectious disease control services under the new antenatal care guidelines: a service availability and readiness assessment of health facilities in Tanzania

**DOI:** 10.1186/s12913-019-3990-8

**Published:** 2019-03-11

**Authors:** Emmanuel Nene Odjidja, Ghislaine Gatasi, Predrag Duric

**Affiliations:** 1 Village Health Works, Bururi, Burundi; 2 Institute of Global Health and Development, Queen Margaret University, Edinburgh, UK

**Keywords:** Infectious diseases, Antenatal care, Service integration, Tanzania, Readiness assessment

## Abstract

**Background:**

Tanzania remains among the countries with the highest burden of infectious diseases (notably HIV, Malaria and Tuberculosis) during pregnancy. In response, the country adopted World Health Organization’s (WHO) latest antenatal care (ANC) guidelines which recommend comprehensive services including diagnostic screening and treatment for pregnant women during antenatal. However, as Tanzania makes efforts to scale up these services under the existing health system resources, it is crucial to understand its capacity to deliver these services in an integrated fashion. Using the WHO’s service availability and readiness assessment(SARA) framework, this study assesses the capacity of the Tanzanian Health System to provide integrated Malaria, Tuberculosis and HIV services.

**Methods:**

Composite indicators of the five components of integration were constructed from primary datasets of the Tanzanian Service Provision Assessments (SPA) under the Demographic and Health Survey (DHS) programs. Chi-squared analysis, T test and ANOVA were conducted to determine the associations of each of the defined components and background characteristics of facilities/health workers. A logistic regression model was further used to explore strength of relationships between availability of service readiness components and a pregnant women’s receipt of HIV, Malaria and TB services by reporting adjusted odds ratios.

**Results:**

Generally, capacity to integrate malaria services was significantly higher (72.3 95% CI 70.3–74.4 *p = 0.02*) compared to Tuberculosis (48.9 95% CI 48.4–50.7) and HIV (54.8 95% CI 53.1–56.9) services. Diagnostic capacity was generally higher than treatment commodities. Regarding the components of SARA integration, logistic regression found that the adjusted odds ratio of having all five components of integration and receiving integrated care was 1.9 (95% CI 0.8–2.7). Among these components, the strongest determinant (predictor) to pregnant women’s receipt of integrated care was having trained staff on site (AOR 2.6 95% CI 0.6–4.5).

**Conclusion:**

Toward a successful integration of these services under the new WHO guidelines in Tanzania, efforts should be channelled into strengthening infectious disease care especially HIV and TB. Channelling investments into training of health workers (the strongest determinant to integrated care) is likely to result in positive outcomes for the pregnant woman and unborn child.

## Background

Tanzania is among countries with leading maternal and child mortality rates in the world. With an estimated under five (U5) mortality rate of 54 deaths per 1000, Tanzania ranks 51st in global U5 mortality rank [1]. Notwithstanding, it has made significant progress in combating neonatal mortality. Between 2000 and 2012, Tanzania experienced a consistent annual decrease rate of 4.3% in neonatal mortality [1]. Afnan-Holmes et al. [2] forecast that if this trend should continue, Tanzania is likely to achieve child health target by 2030.

Regarding maternal health, the United Nations Development Program’s (UNDP) report on the millennium development goals [3] indicates that Tanzania failed to achieve Millennium Development Goals (MDG) target 5.2 as maternal mortality rate stood at 133 deaths per 100,000 live births at 2015. Progress has fallen below targets as annual reduction rate was only 3.4% between 1990 and 2013 [2].

While still births and pregnancy haemorrhage contribute majorly to neonatal and maternal deaths respectively, the impact of infectious diseases especially Human Immunodeficiency Virus (HIV), Malaria and Tuberculosis (TB) during and post pregnancy has been noted by several authors [4–6]. Dreyfuss et al. [6] in a cohort study of 822 HIV positive women found that disease progression to Acquired Immunodeficiency Syndrome (AIDS) and low birth weight were significant outcomes experienced in Tanzania. Similar to this finding, a systematic review by Mullick et al. [7] found that HIV infection during pregnancy in South Eastern Tanzania was significantly associated to chorioamnionitis and low birth weight. As confirmed by previous studies, the prevalence of HIV/TB comorbidity during pregnancy in Tanzania is overwhelming. Mtei et al. [8] in a trial among 94 HIV – positive women found that 71% had been comorbid with TB. Malaria during pregnancy has significant maternal and neonatal outcomes within the Tanzanian context. A study in Northern Tanzania [4] found that not only does malaria increases the risk of anaemia and low birth weight but also, significantly increased the risk of premature delivery (OR 3.2 95% CI, 1.5–7.0).

As a response to the devastating impact of Malaria, TB and HIV infection during pregnancy in this context, the Tanzanian Ministry of Health first adapted and implemented the focused antenatal care model in 2002 [9]. Under this policy, first antenatal care should commence before 16 weeks of pregnancy and should be integrated among other interventions including screening for infectious diseases [9]. Although focused antenatal care (FANC) is intended to mitigate utilisation barriers and improve health of women during pregnancy, challenges around optimal FANC utilisation (4+ visits) largely remain in Tanzania. According to the demographic and Health Survey [10], of the 97.7% women that had first antenatal care, only 42.8% had attained the optimal number of visits (four or more visits). Additionally, median of first antenatal care visit was at 19.7 weeks.

In spite of the lingering health systems challenges which has resulted in lower FANC attendance [11], the Tanzanian Ministry of Health recently agreed to the implementation of the new model of antenatal care. This model requires pregnant women to receive a minimum of eight focused antenatal care visits in lieu of four integrated visits [12].

Ahead of national scale implementation of this new FANC model, we aim to understand the capacity of the health system to deliver an integrated infectious disease (HIV, Malaria and TB) control services in response to the increasing impact of infectious diseases among pregnant women. Furthermore, by adapting the World Health Organisation’s service availability and readiness assessment tool, this study contributes to the limited research of infectious disease control service with antenatal care and further offers technical areas for consideration when scaling integrated infectious disease services with antenatal care.

## Methods

### Study design

The original study was a cross-sectional design conducted among 1200 sample of health facilities, 6866 health workers and 4007 pregnant women across Tanzania. However, for the purposes of this study, variables were constructed from the primary data based on the WHO’s service availability and service readiness assessment (SARA) tool [13] in order to answer the research aim.

### Procedures for variable construction

Secondary variables were constructed as a composite of the primary variables. Based on the original indicators of the SARA tool [13], new variables were re-created for each disease area (HIV, Malaria and Tuberculosis). This included the outcome variable under measure: pregnant woman’s receipt of integrated care. The indicators were;

‘Trained staff’ was constructed as composite of ANC health staff that had recently received training in an infectious disease area. For example, to be classified as technically trained in HIV, the health worker should be the first one that offers ANC service and secondly should be trained in HIV counselling and testing services.

Another indicator, ‘availability of disease service’ was constructed as a composite indicator of having both the ID service and ANC at least three working days within the week. ID service on the same site with ANC was constructed having the disease service at the same place as the antenatal care.

‘Availability of diagnostics’ was classified as a composite indicator of all essential diagnostics of ID service available at the ANC facility. Another indicator, ‘availability of medicines and commodities’ were also constructed as having all essential medicines for each ID service during all times of the ANC facility.

After constructing these individual integration components, a final composite indicator score of all these components was constructed to measure the readiness of facilities to integrate these disease services. This indicator was then disaggregated by disease type.

### Outcome variable

The main outcome variable for the study was receipt of integrated care of infectious disease services (HIV, TB and Malaria). This was based on the hypothesis that if facilities had all five components of integration present, then pregnant women should receive an integrated care during antenatal sessions. The construction of integrated care (a composite indicator) was based on three parameters; first pregnant women’s receipt of insecticide treated nets (ITNs) during antenatal care, second, observation of pregnant women’s consumption of antimalarial (IPTp-SP) during all antenatal sessions after 16 weeks, third was pregnant women’s receipt of HIV counselling and testing during first antenatal care. As guided by the ANC policy guidelines [14], no outcome for tuberculosis was included in this composite outcome variable.

### Data sources

The main source of data for this study was the service provision assessment (SPA) conducted at facilities in both the mainland Tanzania and Zanzibar (the island) in 2015/2016 [15]. The data were collected under the auspices of National Bureau of Statistics (NBS) and the office of the Chief government statistician in Zanzibar and funded by USAID under the demographic and health survey (DHS) program.

The Tanzanian SPA was a survey of availability of services in facilities which spanned from those owned by government, private, parastatal to faith-based organisation (also termed as mission facilities). Also, the survey originally collected information on facilities at all levels of care (i.e. from primary, secondary to tertiary facilities at district, regional and national level). The areas covered during the survey included maternal, neonatal and child health, family planning, sexually transmitted disease services, antenatal care, non-communicable diseases (i.e. cardiovascular diseases, diabetes 1 and chronic respiratory diseases). For each of these disease service areas, the SPA measured availability and functionality of the services which included a quality assessment of equipment, diagnostic tools and essential medicines.

### Data analysis

SPSS (version 20) and Microsoft Excel (2013) were used for all analysis in this study. Five main steps were involved in the analysis. Each of these are explained below.

First, a background characteristics of all ANC facilities and availability of infectious diseases were computed. These were disaggregated by facility type and location.

Second, using the ‘countif’ excel formula, all composite indicators for each ID service area was calculated. The data was then processed in SPSS.

Third, associations and emerging relationships between integration and facility characteristics were explored using an appropriate test. A chi-squared test was used to determine the association between each of the five components of disease integration and background characteristics of facilities.

Fourth, all the five indicators of integration were put together as a single indicator. For pragmatic reasons, this indicator was known as overall integration score for each infectious disease area. This indicator was computed for each of the three disease areas (i.e. malaria, TB and HIV).

Finally, for the association between the components of integration and the outcome variable, a logistic regression model was created to report the odds of association. The model was adjusted for all sociodemographic characteristics and facility/provider characteristics that could have a potential cofounding effect on the results.

Significance level for all tests were pegged at 95% confidence level (i.e. *p* < 0.05).

### Ethical consideration

The original service provision assessment was approved by the National institute for medical research, Tanzania and the macro institutional board of Inner City Fund (ICF) International. According to the primary report of the survey [15], all participants signed a consent form. All participants were fully aware of the implications of engaging in the study including a possibility to have a secondary analysis like this study. The datasets are confidential and do not in any way expose study participants in a manner that affects them. For this particular study, ethical approval was granted by the Institute of Global Health and Development at the Queen Margaret University. For the purposes of protecting identities of the study participants and/or specific facility, all analysis were conducted from district to national level only.

## Results

### Proportion of health workers and facilities that provide antenatal care services and HIV, malaria and TB services

On average, as shown in Table 1, only 21.2% (895/4221) of the health staff provided care in both ANC and disease areas. Assistant medical officers topped the proportion of health staff who provided integrated care with 74.0%. Of all clinical officers and general medical doctors, 62.1 and 57.3% were found to have provided integrated care during antenatal sessions. Unsurprisingly, registered nurses, enrolled nurses and nursing attendants had the least proportion of staff who provided integrated care. Chi-squared analysis showed that there were significant differences among cadres of staff. This result was expected as there were a 67.4% difference between the highest and lowest proportions.

**Table 1:** Proportion of ANC staff that offer integrated service in HIV, Malaria and TB

Proportion of health workers who provide ANC service together with both HIV, Malaria and TB services	*N*	%	Sig level	Value	df
*Disaggregated by staff cadre*			< 0.024	5236.3	52
General Medical Doctor	51	57.3			
Specialist Medical Doctor	10	26.3			
Assistant Medical Officer	145	74.0			
Clinical Officer	239	62.1			
Assistant Clinical Officer	40	43.0			
Registered Nurses	148	13.1			
Enrolled Nurse	203	13.5			
Nurse Assistant/Attendant	51	6.6			
Other cadres	8	27.6			
Total	895	21.1 (895/4221)			

Similar to provision of integrated services by providers, facilities that reported of having integrated services in HIV, Malaria and TB were generally low across all areas. Table 2 shows that on overall, 48.2% reported of having all services with facilities in urban areas reporting 64.0% in contrast to 41.4% in rural areas. As nurses and midwives (who reported the least of providing integrated care) predominantly work in rural areas, it is unsurprising to find that rural facilities were significantly lower (*p* < 0.001) in having HIV, Malaria and TB services than urban facilities. Similarly, as most nurse assistants (who reported only 6.6% having offered integrated care) are found in dispensaries, it is least striking that dispensaries reported of just 8.8% having integrated services.

**Table 2:** Proportion of ANC health facilities that offered integrated services in Malaria, HIV and Tuberculosis

Proportion of ANC facilities that have HIV, Malaria and TB services	*N*	%	Sig level	Value	df
*Disaggregated by managing Authority*			0.011	471.1	15
Government/Public	351	46.6			
Private-for-profit	20	25.0			
Mission/Faith-based facilities	115	62.2			
Parastatal facilities	11	91.7			
*Disaggregated by Facility location*			< 0.001	153.1	5
Rural	298	41.4			
Urban	199	64.0			
*Disaggregated by facility type*			0.031	794.5	35
National Referral Hospital	5	71.4			
Regional Hospital	16	94.1			
District Hospital	73	98.6			
District designated hospitals	20	100			
Other Hospital (Private)	96	83.5			
Health Centre	250	69.6			
Dispensary	37	8.8			
Total	497	48.2 (497/1031)			

### Overall integration capacity for each disease area

To measure integration of all disease areas based on the SARA tool, a standardised score of all components on a scale of 100 was computed to determine the overall readiness integration capacity of facilities in the three disease areas.

Results as shown in Table 3 found that malaria control obtained the highest score (72.3 95%CI 70.3–74.4) followed by HIV/AIDS and then Tuberculosis control. This insinuates that the current readiness is stronger in malaria followed with HIV/AIDS then to Tuberculosis control. It is unsurprising as components of malaria integration were widely present in most ANC facilities.

**Table 3:** Overall integration capacity of health facilities in HIV, Malaria and TB

Overall disease area integration capacity	Standardised score (computed on a scale of 100)	95% CI
Tuberculosis control integration score	48.9	48.4–50.7
HIV/AIDS control integration score	54.8	53.1–56.9
Malaria control integration score	72.3	70.3–74.4

### Association between integration capacity and receipt of integrated care during antenatal care

The main outcome variable of study was receipt of integrated service (defined as receipt of comprehensive HIV, TB and Malaria services during antenatal care) and the odds with which a pregnant woman would receive integrated services should all the components of these diseases be present in a health facility.

Results from a logistic regression model as displayed in Table 4 found that if all components for service integration were present for all diseases, a pregnant woman was 1.9 times (95% CI 0.8–2.7) likely to receive a comprehensive and integrated care. This therefore confirms that there is a positive relationship between integration capacity and receipt of integrated services. Furthermore, when the model was disaggregated into the five components of integration, it found availability of a trained staff during antenatal care as the strongest predictor to receipt of integrated services (AOR 2.6 95% CI 0.6–4.5). Facilities with all recommended diagnostics were 1.4 times (AOR 1.4 95% CI 1.1–3.2) more likely to administer integrated care during ANC sessions. The adjusted odds of administering integrated care in facilities that had essential medicines and treatment commodities was 1.1 (95% CI 0.9–2.3). Facilities with disease service available at least three times a week were 1.3 times likely (95% CI 0.9–2.4) to administer integrated care for pregnant women during ANC sessions.

**Table 4:** Logistic regression of integration components and receipt of integrated services

Component of integrated service	AOR	95% CI	Sig level
Availability of all integration components (for HIV, TB and Malaria)	1.9	0.8–2.7	0.011
Availability of diagnostics for all disease areas	1.4	1.1–3.2	0.147
Availability of medicines and treatment commodities	1.1	0.9–2.3	< 0.001
Availability of trained staff in all disease areas	2.6	0.6–4.5	0.002
Disease service and antenatal care situated at the same site	1.0	–	–
Disease service available at least three times a week during antenatal care	1.3	0.9–2.4	0.033

Finally, the model found that having disease service at the same site was not enough to receive integrated services as the adjusted odds was one (no effect).

## Discussion

The aim of the study was to assess service readiness of Tanzanian health facilities to integrate HIV, Malaria and Tuberculosis under the new recommendations of the World Health Organisation. This also included the extent to which the components of service integration resulted in actual service delivery. Based on the SARA framework, components of service integration was constructed under five main domains. Findings from this study has demonstrated that having all components of service integration were significantly associated with receipt of integrated service during antenatal care. Among the five components of integration, the study found that training an ANC health worker in all disease areas were the strongest determinant to receipt of an integrated service during ANC sessions.

The conceptualisation of receipt of integrated care during antenatal care in this study was based on five set parameters. The adjusted odds of having these parameters and receipt of integrated care were determined as having a positive relationship. Based on these findings, this study supports Foreit et al. [16]‘s assertion that the prime determinant of integrated care during antenatal care is having trained staff on site. It was found that the adjusted odds of having a trained staff on site and receipt of integrated care by pregnant women were 2.6 times and up to 4.5 times during antenatal care. While it is inadequate to claim complete integration based on just availability of trained staff, it is an important and key step in ensuring that pregnant women receive all the requisite HIV, Malaria and TB services. Towards the implementation of the new WHO ANC guidelines, it is relevant for Tanzanian public health planners to consider training all health workers in all disease areas.

The significance of training health staff in disease areas have been noted by several authors. Ouma et al. [17] noted that all components of malaria service delivery were higher among facilities that had health staff engaged in a donor funded training in malaria. Furthermore, the authors contend that training a health worker in malaria does not merely improve service delivery, but it also improves pregnant women’s experience during antenatal care which eventually improves coverage of future ANC services [17]. Welty et al. [18] also observed that HIV voluntary screening and counselling had increased significantly from 50.7 to 96.2% (*p* > 0.01) after training health workers on HIV care.

Although organising on-the-job training programs has been recognised as a key determinant to successful ‘training’ and knowledge transfer, its sustainability would be assured when backed with quality improvement procedures [19]. Batalden and Davidoff [19] identifies three quality improvement areas which could complement routine trainings of health workers. These are; 1. Generating contextual evidence to guide training of health workers, 2. Routine capacity building and 3. Consistent measurement of performance improvement.

Generating contextual evidence during trainings of health staff ensures that context-specific issues are incorporated into training programs. Context-tailored trainings responds to local needs and demands of health workers. In addition, Batalden and Davidoff [19] argues that incorporating routine on-the-job capacity building strengthens quality improvement and further ensures that policy and practice are consistent during all times of service delivery. Finally, creating mechanisms to measure performance of health workers before and after training programs would ensure that individual health worker capacity is taken into consideration during planning and targeting of training programs. Effective performance measurement also ensures that subsequent capacity building programs are tailored to respond to identified areas where majority of health workers may deem as a ‘weakness’. Allen and Brownstein [20] on an assessment of community health workers’ training programs in the United States reinforced the significance of incorporating effective performance measurement before and after training programs. They further recommend that adding qualitative exit interviews strengthens quality improvement even more.

### Study limitations

The study encountered some limitations which were noted during the data analysis. Firstly, the nature of original service provision assessment survey being cross-sectional limits this study from claiming causality of receipt integrated care during antenatal session. This is due to the fact that construction of the components of integration were based on a combination of individually collected variables. In this case, when a pregnant woman receives several ID services from the same tertiary facility in different sections for example, the nature of analysis will classify it as vertical services. However, although this is likely to occur only among the five referral hospitals, a confidence interval (95%) range was calculated for all results. This was intended to reduce the impact of this limitation by computing the minimal and maximal boundaries upon which results could be estimated.

## Conclusion

This study has argued that integration of health services is an effective mechanism to increase coverage and to reduce the impact of infectious diseases during pregnancy. Using the SARA framework, it was found that the strongest determinant of receiving integrated care during antenatal session was having a trained staff in all disease areas on site. Unexpectedly, the model found that having antenatal care services and ID service on the same site had no association to receiving integrated care.

Towards readiness to integrate infectious disease control with antenatal care services under the new recommendations, strengthening capacity of health workers to administer this care will be crucial in higher coverage of services. Supportive quality improvement is crucial in this process and as such incorporating different techniques for continuous support to health workers ensures latest policy and practice are consistent. As illustrated by Batalden and Davidoff, driving this process with evidence even yields maximum benefits on the health system as a whole. Health workers are central to the process of supportive quality improvement and it is relevant to create a down-to-top heath needs assessment that continuously informs capacity building sessions.

Finally, while complete integration of ID services at ANC may seem distant in Tanzania, health authorities should take the initial short term step of effectively co-locating these services according to the identified determinants. As lessons have shown, the nuanced effectiveness of this strategy will gradually encourage other key health actors to pursue overall integration and devolution of health services.

## Abbreviations

ANC: Antenatal Care
ANOVA: Analysis of Variance
AOR: Adjusted Odds Ratio
DHS: Demographic and Health Survey
FANC: Focused Antenatal Care
HIV: Human Immunodeficiency Virus
ICF: Inner City Fund
ID: Infectious Disease
MDGs: Millennium Development Goals
OR: Odds Ratio
SARA: Service Availability and Readiness Assessment
SPA: Service Provision Assessment
SPSS: Statistical Package for the Social Sciences
TB: Tuberculosis
U5: Under 5
UNDP: United Nations Development Program
USAID: United States Agency for International Development
WHO: World Health Organisation
