# Correction to: Delivery of integrated infectious disease control services under the new antenatal care guidelines: a service availability and readiness assessment of health facilities in Tanzania

**DOI:** 10.1186/s12913-021-06588-w

**Published:** 2021-07-09

**Authors:** Emmanuel Nene Odjidja, Ghislaine Gatasi, Predrag Duric

**Affiliations:** 1 Village Health Works, Bururi, Burundi; 2 Institute of Global Health and Development, Queen Margaret University, Edinburgh, UK

**Correction to: BMC Health Serv Res 19, 153 (2019)**

**https://doi.org/10.1186/s12913-019-3990-8**

Following publication of the original article [1], the authors would like to make the following changes:

1. The methodology described in the abstract needs to be corrected to eliminate T test and ANOVA as both tests were not used in the main methods.

The sentence currently reads:

Chi-squared analysis, T test and ANOVA were conducted to determine the associations of each of the defined components and background characteristics of facilities/health workers.

The sentence should read:

Chi-squared analysis was conducted to determine the associations of each of the defined components and background characteristics of facilities/health workers.

2. The last paragraph under the background needs to include a text of the initial study and cite it accordingly

The last paragraph reads:

Ahead of national scale implementation of this new FANC model, we aim to understand the capacity of the health system to deliver an integrated infectious disease (HIV, Malaria and TB) control services in response to the increasing impact of infectious diseases among pregnant women. Furthermore, by adapting the World Health Organisation’s service availability and readiness assessment tool, this study contributes to the limited research of infectious disease control service with antenatal care and further offers technical areas for consideration when scaling integrated infectious disease services with antenatal care.

This should read:

Ahead of national scale implementation of this new FANC model, we aim to understand the capacity of the health system to deliver an integrated infectious disease (HIV, Malaria and TB) control services in response to the increasing impact of infectious diseases among pregnant women. This builds on a previous study which study which employed the World Health Organisation’s service availability and readiness assessment tool and defined integration based on five indicators in the context of Kenya, Malawi and Tanzania [13]. A value addition of this study is an assessment whether availability of integrated care results in receipt of integrated service from health facilities under the latest ANC recommendations in Tanzania.

3. The study design under the methods section needs to include a citation of the original work.

The sentence currently reads:

The original study was a cross-sectional design conducted among 1200 sample of health facilities, 6866 health workers and 4007 pregnant women across Tanzania. However, for the purposes of this study, variables were constructed from the primary data based on the WHO’s service availability and service readiness assessment (SARA) tool [14] in order to answer the research aim.

This paragraph should read:

The original study was a cross-sectional design conducted among 1200 sample of health facilities, 6866 health workers and 4007 pregnant women across Tanzania. However, for the purposes of this study, variables were constructed from the primary data based on the WHO’s service availability and service readiness assessment (SARA) tool [14] and using indicators of integration as offered in a previous study of Mallick et al. [13].

4. Under the section ‘Procedures for variable construction’, some changes need to be made.

The text beneath this section currently reads:

Secondary variables were constructed as a composite of the primary variables. Based on the original indicators of the SARA tool [13], new variables were re-created for each disease area (HIV, Malaria and Tuberculosis). This included the outcome variable under measure: pregnant woman’s receipt of integrated care. The indicators were;

This paragraph should read:

Secondary variables were constructed as a composite of the primary variables via adopting and adapting indicators of integration as defined by Mallick et al. [13]. This included the outcome variable under measure: pregnant woman’s receipt of integrated care;

5. Citation of Mallick et al. [13] need to accompany some indicators.

The statement currently reads:

Another indicator, ‘availability of disease service’ was constructed as a composite indicator of having both the ID service and ANC at least three working days within the week.

This statement should read:

Another indicator, ‘availability of disease service’ was constructed as a composite indicator of having both the ID service and ANC at least three working days within the week [13].

Another indicator currently reads:

ID service on the same site with ANC was constructed having the disease service at the same place as the antenatal care.

This statement should read:

ID service on the same site with ANC was constructed having the disease service at the same place as the antenatal care [13].

Finally, the indicator on availability of diagnostics currently reads:

‘Availability of diagnostics’ was classified as a composite indicator of all essential diagnostics of ID service available at the ANC facility.

This should read:

‘Availability of diagnostics’ was classified as a composite indicator of all essential diagnostics of ID service available at the ANC facility [13].

6. The discussion needs to discuss consistency in findings between this study and that of Mallick et al. [13]

The paragraph currently reads:

Among the five components of integration, the study found that training an ANC health worker in all disease areas were the strongest determinant to receipt of an integrated service during ANC sessions.

It should read:

Among the five components of integration, the study found that training an ANC health worker in all disease areas were the strongest determinant to receipt of an integrated service during ANC sessions. Consistent with this finding, Mallick et al. [13] found that having ANC providers, trained in an infectious disease area providing for care in a health facility significantly predicted receipt of care during pregnancy.

13. Mallick, Lindsay, Rebecca Winter, Wenjuan Wang, and Jennifer Yourkavitch. 2016. Integration of Infectious Disease Services with Antenatal Care Services at Health Facilities in Kenya, Malawi, and Tanzania. DHS Analytical Studies No. 62. Rockville, Maryland, USA: ICF International.
